# Long bones after suspected “grave robbery”: a comparison of different methods for the estimation of the post mortem interval

**DOI:** 10.1007/s12024-025-01140-2

**Published:** 2025-11-27

**Authors:** Johannes Baumgarten, E. Stephan, T. E. N. Ohlwärther, F. Holz, C. G. Birngruber, M. A. Verhoff, P. J. Chabiera, S. C. Kölzer

**Affiliations:** Institute of Legal Medicine, Goethe University Frankfurt, University Hospital, Kennedyallee 104, Frankfurt am Main, 60596 Germany

**Keywords:** Forensic osteology, PMI, Human bones, UV-fluorescence – luminol

## Abstract

Estimation of the postmortem interval (PMI) of found bones is an important and challenging part of forensic osteology assessments. This study examined human long bones that had been taken from cemeteries and hoarded by a "bone collector". Based on the police investigation and own investigation into the length of grave leases in the pertinent cemeteries, the narrowed down PMI for the bones was between 20 and 100 years. Our aim was to evaluate the suitability of the UV-fluorescence and luminol methods in determining the PMI of these bones and to assess the reliability of the results for forensic practice. Based on macroscopic criteria, 201 bones were classified into various PMI groups. Freshly sawn bone surfaces were then assessed with UV-fluorescence and luminol. The UV-fluorescence examination showed a weak to mediocre correlation between the intensity of UV-fluorescence and the PMI estimated by macroscopic criteria. Surprisingly, the luminol test did not reveal a negative correlation between the degree of chemiluminescence and macroscopically estimated PMI. Within a PMI span of up to 100 years, the extent of UV-fluorescence can serve only as a rough indicator of PMI. Alone, the method does not suffice to identify forensically relevant PMIs. Likewise, the luminol test does not reliably distinguish between bone finds with and without forensically relevant PMI. Nonetheless, the assumption that a negative luminol-test still speaks for a historical find appears to be justified, and, at least in combination with other tests, the luminol test can be used.

## Key points

• The UV-fluorescence examination can help to divide bone finds into the categories “forensic relevant” and “not forensic relevant”.

• A reduction of the blue UV-fluorescence can indicate a lack of forensic relevance, but false negative results can occur.

• The luminol test cannot reliably distinguish between bone finds with or without forensically relevant PMI.

• To get the most reliable outcome, a combination of the aforementioned methods in particular with the macroscopic examination can be recommended.

## Introduction

The estimation of the postmortem interval (PMI) for bone finds is an important and challenging part of expert forensic osteology assessments. The present study examined human long bones that had been stolen from cemeteries and then hoarded by a “bone collector”. These cemetery bones formed the collective for the current study. Based on the results of the police investigation and knowledge of the burial customs and practices at the cemeteries in question, a PMI range for the collected bones could be estimated.

### Funeral culture and law

Funeral culture is diverse and deeply rooted in religious and cultural traditions, which significantly influence funeral and burial practices. In Judaism, Christianity, and Islam, for example, interment (also known as inhumation) has been the most common form of burial for thousands of years. Additionally, burial practices and procedures may be regulated by law. In Germany, for instance, each of the federal states has its own burial laws, all of which include an obligation to bury or cremate the decedent. Since 1934, the burial laws in most German federal states also stipulate that human remains or ashes must be laid to rest in cemeteries [1]. In addition, minimum mandatory grave lease lengths, respectively rest periods, are imposed to ensure decomposition of the body before reuse of the gravesite. Depending on the capacity and soil conditions in each cemetery, these rest periods may vary across cemeteries, whereby these conditions, of course, significantly affect the decomposition period of a corpse [2]. The minimum grave lease length in most German federal states is, on average, 20 to 30 years, whereby the respective rest period in each cemetery is determined by factors like soil texture, age at death of the decedent, and burial type. In exceptional cases, graves may also be left in place indefinitely, with an unlimited rest period; for example, in the case of state burials, or those to which special religious or socio-cultural considerations apply, as in Jewish cemeteries.

### The bone discovery

In 2018 and 2019, there was an increase in urn thefts at cemeteries in Frankfurt am Main and surrounding areas. After a suspect had been identified by the police, countless bones had been found in his flat alongside urns, grave slabs and grave goods. The police investigation revealed that the “bone collector” had stolen these items from various cemeteries in the Frankfurt metropolitan area over a period of several years, presumably from earth mounds from graves that had been legally excavated after the gravesite leases had expired. The "bone collector" had taken the unearthed bones to his flat, where he had kept them until they had been confiscated by the police. Later, the bones were handed over to the Institute of Legal Medicine in the numerous boxes and bags in which they had been stored. In these, more than 3,000 bones and bone fragments of predominantly human, but also of animal origin were found, in addition to grave goods and other non-organic components. Further research conducted by the authors of this study into the mandatory grave lease lengths as well as possibilities of extending grave leases at the respective cemeteries revealed that the PMI of the seized bones could, at maximum, have been around 100 years. Further background information on this case can be found in the recently published case report [3]. Prior to our study, the responsible public prosecutor’s office had withdrawn their call for forensic-osteological examination of the bones, which had, in particular, been ordered to clarify the question of a forensically relevant post-mortem interval [4]. The bones, which were subsequently released for scientific study, formed the sample collective for the present study.

### The significance of postmortem interval from the perspective of criminal law

The determination or estimation of the postmortem interval (PMI), also known as the time since death, from skeletal remains has always been one of the greatest challenges in forensic osteology. Basically, the PMI is defined as the time span between the time of death and the time of the examination [5]. The main problem in determining this time span lies in the great complexity of external influencing factors. These factors are often almost impossible to calculate, even if the bones have continuously lain in the ground. Furthermore, in individual cases, these factors can also influence each other [6]. Even if the investigation is limited to distinguishing between a historical and a recent, respectively forensically relevant, bone find, estimating the PMI proves to be difficult. To date, only radionuclide methods have been able to fulfill the requirement of determining the PMI independently of external factors [7–10]. However, due to disadvantages, especially like high costs and a long examination time, and because they are still not suitable particularly for bones with a short PMI, radionuclide methods have not yet found their way into everyday forensic practice. For this reason, there is still incentive to establish cost-effective and easy-to-use examination methods for PMI determinations. Despite their methodological limitations, macroscopic examination of the bone and assessment of a freshly sawn cross-section with concomitant examination of the cross-section for UV-fluorescence [11] are the first steps taken in determining PMI. These examinations can be backed up with the luminol test. Although this test is not considered sufficient as the sole method, on the basis of previously published studies, it is, in combination with other methods, still considered to be quite helpful in assessing the PMI [12–16].

Based on the results of the police investigation and our own research in regard to the minimum grave lease lengths in the pertinent cemeteries, the PMI span for the bones that were confiscated from the apartment of the “bone collector” was presumed to be between 20 and 100 years. With this collection of skeletal remains a larger number of bone samples with a PMI distribution that can be described as largely homogeneous thus became available for testing with the PMI methods commonly used in forensic osteological practice [17] (macroscopy, UV-fluorescence, luminol test).

The aim of the present study was to evaluate the suitability of the UV-fluorescence method and the luminol test in determining the PMI of these bones and to assess the reliability of these methods for forensic practice.

## Materials and methods

After the bones had been seized and transferred to the Institute of Legal Medicine in Frankfurt, they were stored there in a dark, dry evidence chamber at around 18 °C for a period of around two years until the start of the present study. From the more than 3,000 bones and bone fragments in this collective, all femora, tibiae, and humeri, or parts thereof, were initially selected for the present study. At first, bone fragments had also been considered, if they could be reliably assigned to one of these bones. A total of 392 bones could initially be included in the present study.

To enable permanent identification, the selected bones were labelled with a combination of a randomly assigned serial number, the number assigned by the police to each box or bag (No. 1–17), and the number of smaller bags in the boxes containing the bones. The bones were then macroscopically categorized into four degrees of damage:

- Grade 1: No damage.
- Grade 2: Minor damage (shaft largely preserved in combination with both articular surfaces).
- Grade 3: Major damage (only one articular surface, but in combination with a larger part of the shaft).
- Grade 4: Fragments (only parts of one of the two articular surfaces with parts of the shaft, or isolated parts of the shaft).

In the following, out of the 392 initially included bones, only such with damage grades 1 to 3 were analyzed further. The macroscopic assessment of the fragments in group 4 was considerably more difficult than in the other categories and further processing, in particular, making a saw cut on the fragments, was not considered feasible. Thus, 201 bones remained for further analyses.

In view of the results of the police investigation, according to which the “bone collector” had stolen the bones from cemeteries, it could be assumed that the found bones had lain in the ground for, by far, the largest part of their postmortem interval. The first step was therefore macroscopic assessment of the bones with regard to the postmortem interval, using the morphological criteria developed by Verhoff et al. [11, 18].

Based on the macromorphological criteria according to Verhoff et al. [5, 11] and with the forensically relevant period of 50 years established in forensic practice in Germany in mind [4, 19], the bones were divided into the following categories:

- Group 1: PMI clearly < 50 years.
- Group 2: PMI rather close to < 50 years (≤ 50).
- Group 3: PMI rather close to > 50 years (≥ 50).
- Group 4: PMI clearly > 50 years.

For the further analyses (UV-fluorescence of a freshly sawn cross-section and luminol test), a fresh saw cut was made on the shaft of the long bones using a medical bone saw, after the bone surface had been cleaned with emery paper. The resulting bone meal was collected on a fresh sheet of white A4 paper and transferred to a sample tube for the luminol test.

The assessment of the blue UV-reflection of the sawn cross-sections of the bones was carried out in a darkened room using an UV-lamp with a wavelength of 365 nm. Based on previous studies [15, 19], the extent of blue reflection was divided into the following categories:

- Complete.
- Patchy reduction.
- Sandwich effect (= ring-like reflection of the internal compacta component).
- Spotty residues.
- No reflection.

The luminol test was carried out in a completely darkened room. For this purpose, 50 mg of each bone meal sample was weighed using a precision scale and then placed in prepared test tubes, each containing 5 ml of a luminol solution, freshly prepared according to Weber [20], sealed with a screw cap, and then shaken vigorously for a few seconds. The intensity of the chemiluminescence was then immediately assessed, whereby the intensity was categorized analogously to other studies [15, 16, 19] as follows:

- negative (-).
- weakly positive (+).
- positive (++).
- strongly positive (+++).

The assessment was always carried out by two investigators (JB and ES), independently of each other. The positive control was performed with a drop of human blood that showed maximum fluorescence (+++) in 5 ml of luminol solution.

## Results

The 201, ultimately examined bones consisted of 80 femora, 52 humeri, and 69 tibiae. In almost all cases, it was possible to assign the bones to a body side: 87 (43.3%) of the bones could be assigned to the left side of the body, and 113 (56.2%) to the right side. Only one bone, which was more severely damaged, could not be assigned to a body side. As far as possible, sex discrimination was also carried out using macroscopic criteria, whereby 115 bones (57.2%) could be assigned to the male sex, and 76 (37.8%) to the female sex. For 10 bones (5.0%), no reliable sex discrimination was possible; these bones, however, belonged, in equal numbers, to the categories with minor or more severe damage.

### Macroscopy

**Fig. 1:**
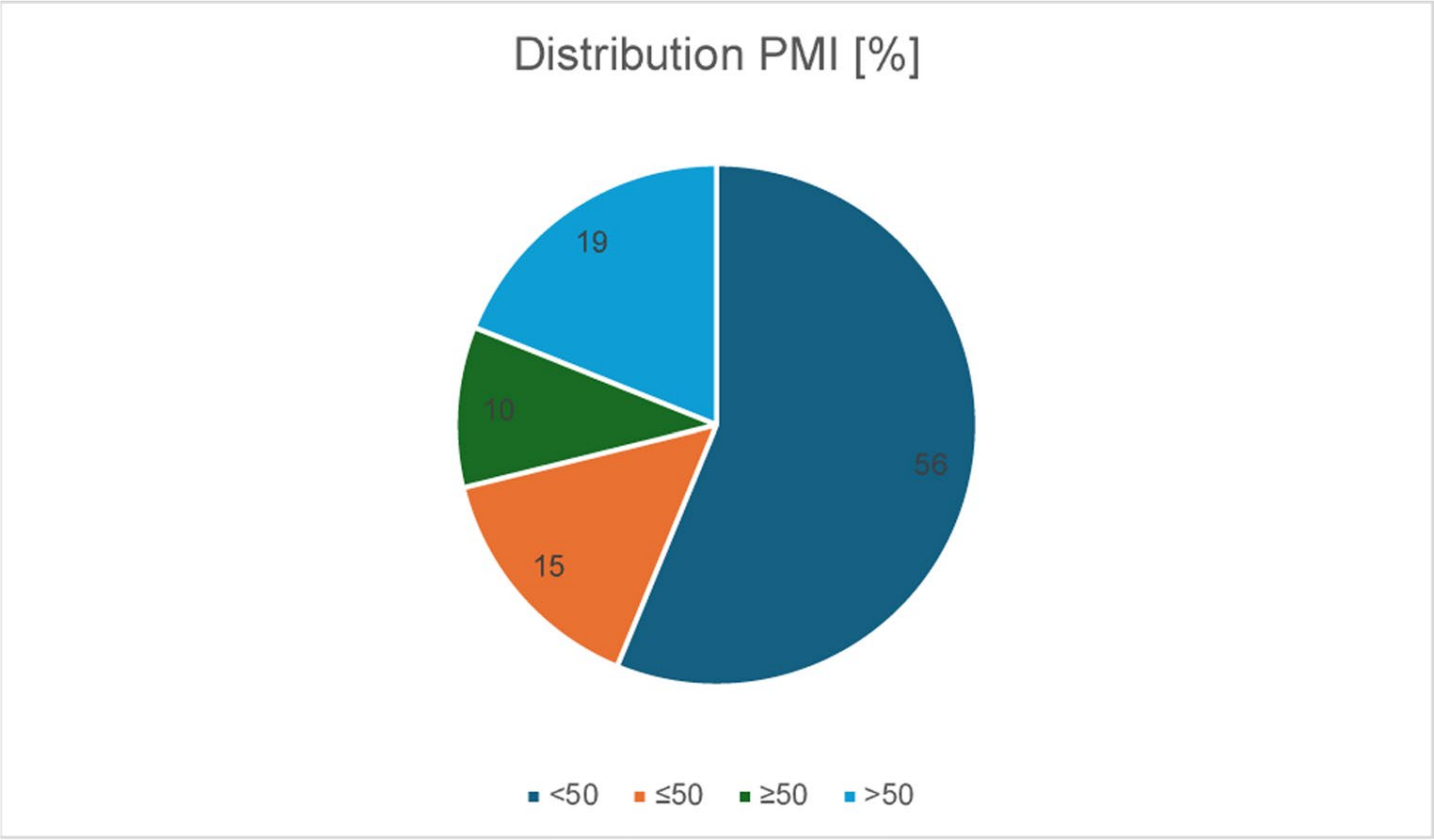
Percentage distribution of the bones among the various PMI groups

Based on the macromorphological criteria according to Verhoff et al. [5, 11] and the forensically relevant period of 50 years established in forensic practice [4, 19], the bones were divided into the following categories:

The percentage distribution of the bones among the various PMI groups is shown in Fig. 1.

### UV-fluorescence

**Fig. 2:**
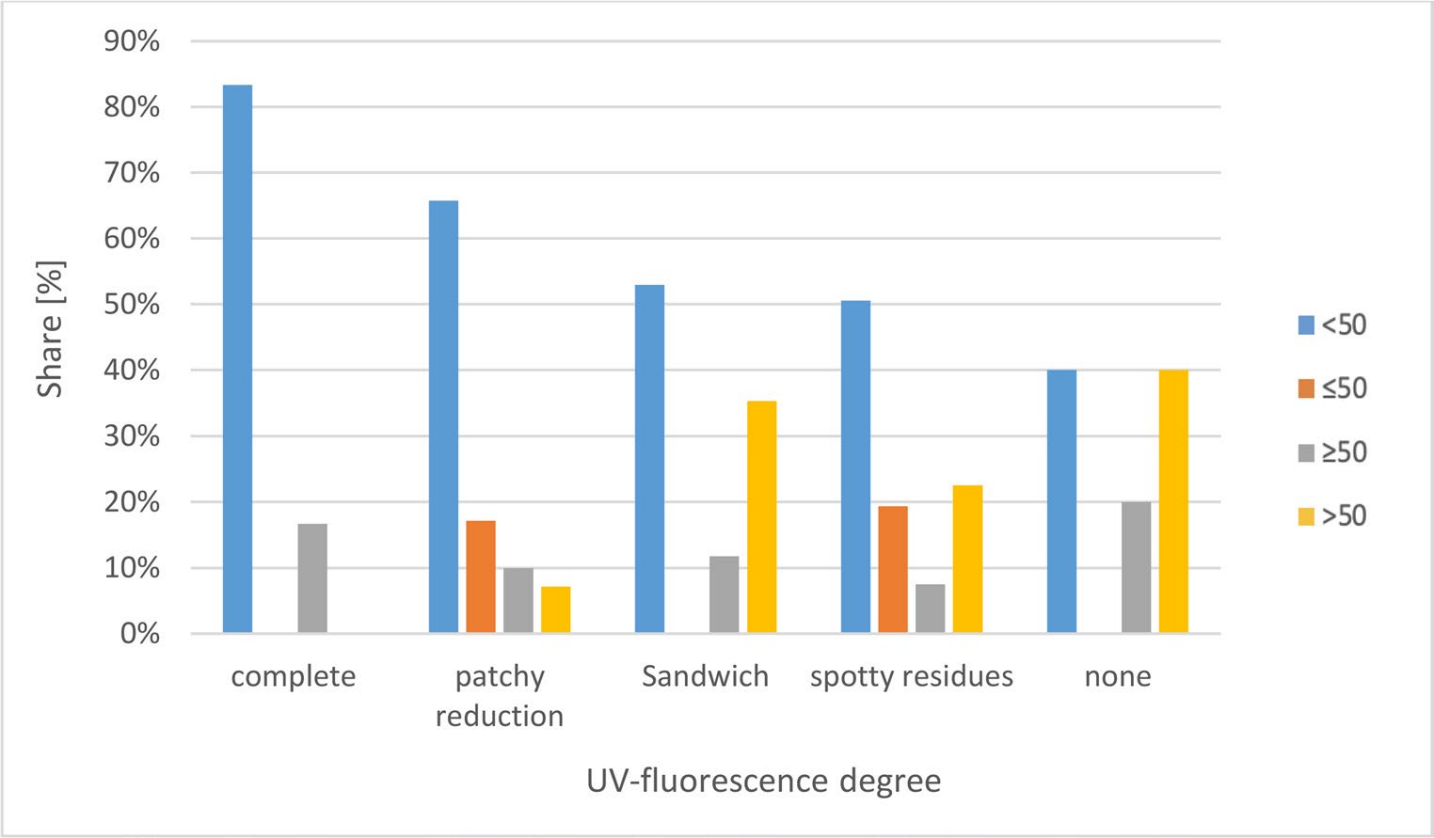
UV-fluorescence as a function of the macroscopically estimated PMI

The results of the UV-fluorescence levels as a function of the macroscopically estimated PMI are shown in Fig. 2; Table 1.

**Table 1:** Percentage distribution

UV-fluorescence	PMI Group			
	< 50	≤ 50	≥ 50	> 50
Complete	83.3%	0%	16.7%	0%
Patchy reduction	65.7%	17.1%	10.0%	7.1%
Sandwich	52.9%	0%	11.8%	35.3%
Spotty residues	50.5%	19.4%	7.5%	22.6%
None	40.0%	0%	20.0%	40.0%

A correlation between the two ordinal variables UV-fluorescence level and PMI group was determined both with a Spearman correlation analysis (g = −0.22, *p* < 0.01) and with the gamma test (g= −0.3; *p* < 0.01).

### Chemiluminescence of the luminol test

**Fig. 3:**
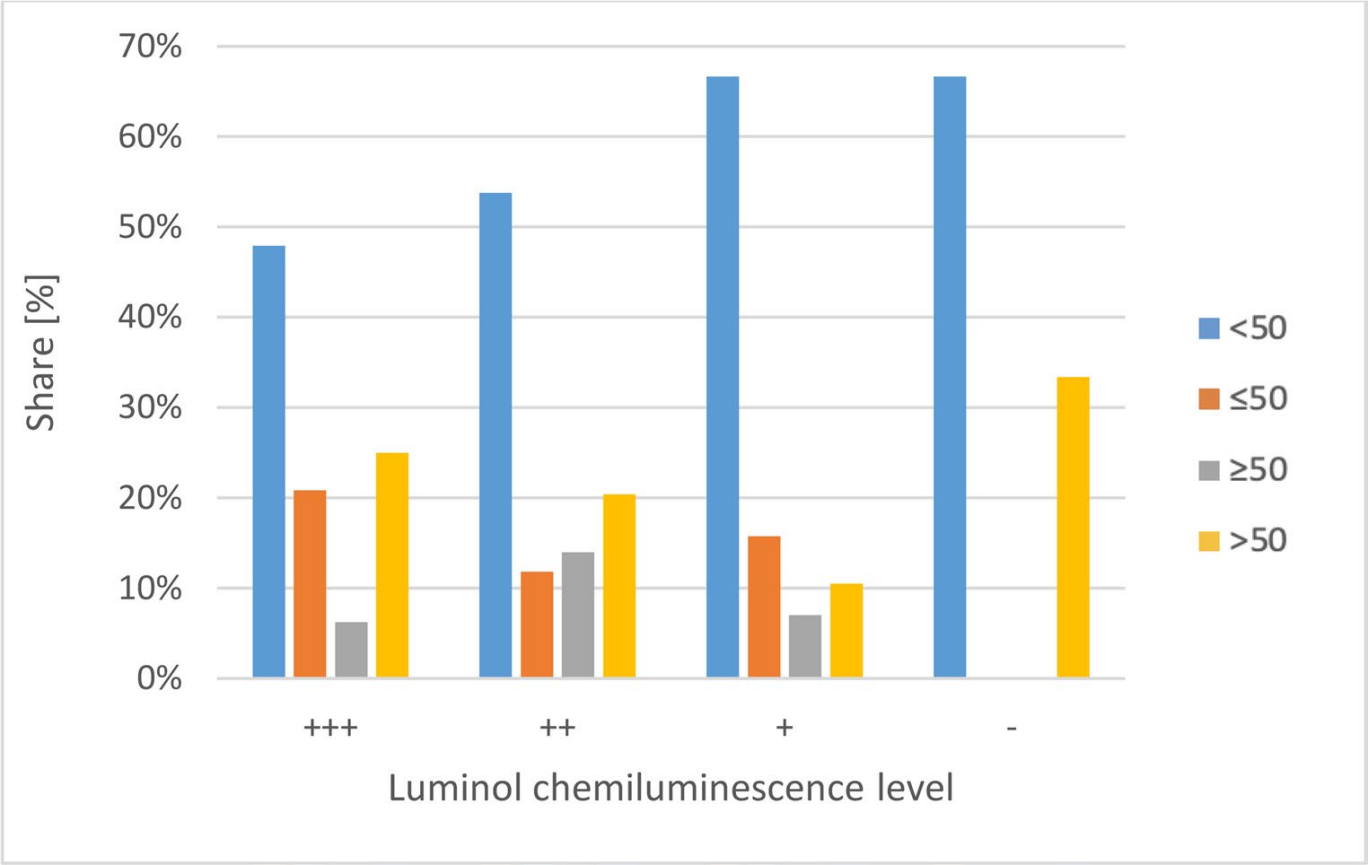
Degree of chemiluminescence of the luminol test as a function of the macroscopically estimated PMI

The results depending on the various PMI groups are shown in Fig. 3; Table 2.

**Table 2:** Percentage distribution

Luminol chemiluminescence level	PMI Group			
	< 50	≤ 50	≥ 50	> 50
+++	47.9%	20.8%	6.3%	25.0%
++	53.8%	11.8%	14.0%	20.4%
+	66.7%	15.8%	7.0%	10.5%
–	66.7%	0%	0%	33.3%

A correlation between the two ordinal variables luminol chemiluminescence level and PMI group was determined both with a Spearman correlation analysis (g = 0.15, *p* < 0.05) and with the gamma test (g = 0.21; *p* < 0.05).

The inter-observer test revealed a high degree of agreement in the assessment of the intensity of chemiluminescence by the two investigators (JB and ES) (Cohen’s kappa = 0.8; *p* < 0.01).

## Discussion

In the present study, a total of 201 human long bones (humeri, femora, tibiae) were subject to PMI determination. Presumably, the bones had lain in the ground during the PMI until they were legally unearthed after expiration of the grave leases. They were then discovered and taken by a “bone collector.” Although the exact PMI of the respective bones was unknown, it could be narrowed down to a likely range of 20 to 100 years, based on the results of the police investigation and our own investigation, especially after evaluating the valid laws, the regulations of the cemeteries and the age of the oldest graves on the relevant parts of the cemeteries. However, it should be noted that we cannot exclude a PMI of slightly more than 100 years of individual respectively a small number of the examined bones.

The bones had then presumably been stored in the home of the “bone collector” during the period between their discovery, or removal from the respective cemeteries, and their confiscation by the police. From the authors’ point of view, this period of time, as well as the subsequent storage period of around two years at the Institute of Forensic Medicine, should not be counted towards the PMI, as it can be assumed that the decomposition process did not proceed significantly under these conditions.

The bones were assessed in regard to their macroscopic appearance, UV-fluorescence of a freshly sawn cross-section, and the chemiluminescence intensity of the luminol reaction of the bone meal obtained when setting the saw cut.

The extent of UV-fluorescence on the freshly sawn cross-section showed a weak to, at best, moderate, significant correlation with the macroscopically estimated PMI. These results fuel the disagreement arising in previous studies as to whether UV-fluorescence is an appropriate method to determine PMI, or whether it even provides valid results. Basically, for decades, UV-fluorescence has been seen as an effective way of assessing PMI, particularly in regard to the exclusion of a forensically relevant period [11, 15, 19, 21–23]. In light of the fact that the exact cause for UV-fluorescence of bone is still largely unknown, this circumstance seems all the more astonishing. So far, the results from various studies indicate that the protein component of the collagen in the bone substance could be responsible for the blue fluorescence in relatively fresh bone samples [24, 25], while, with increasing PMI, the change in mineral content with a simultaneous decrease in the collagen content could explain the rather yellowish to brownish fluorescence of older bone material [26]. In a more recent study by Hoke et al. [27], both the intensity and the color of bone UV-fluorescence in relation to PMI were investigated in a sample consisting of 58 cemetery bones with a known PMI between 8 and 60 years - which roughly corresponds to the PMI range of the bone collective in our present study. Because no significant correlation between the extent of UV-fluorescence and PMI was found by Hoke et al., they regarded this method of differentiating between recent and historical bones to be doubtful. On the other hand, they were able to establish a weak correlation between the color of the UV-fluorescence and the PMI. Similarly, Sterzik et al. were also able to demonstrate a significant correlation between the extent of fluorescent UV-light and 490 nm light, respectively, the color of fluorescence and the PMI [28, 29]. The correlation found by Sterzik et al. was, however, stronger than that found Hoke et al. [27].

Our investigations showed that with (macroscopically estimated) increasing PMI, there was a significant increase in the proportion of bones that showed only slight or no blue fluorescence of the freshly sawn cross-section under UV-light, and none of the bones from the PMI group 4 showed complete UV-fluorescence. In contrast, the finding that 6 of the 15 bones in PMI group 4 still showed at least patchy remnants of UV-fluorescence suggests that already a reduction in blue fluorescence may indicate a PMI beyond forensic relevance and complete absence of UV-fluorescence is perhaps not required. This finding is also consistent with the results from studies by Facchini et al. [22] and Hoke et al. [27], who were still able to detect remnants of blue UV-fluorescence in bones that were demonstrably several hundred to over 10,000 years old. These results even led Hoke et al. to conclude that it is impossible to specify an upper PMI limit for the occurrence of blue fluorescence. Furthermore, Hoke et al. discuss several factors that can significantly influence fluorescence intensity: The infiltration of Mn^2+^ or Fe^2+^ ions could, for example, reduce fluorescence; hence, the burial environment could play a decisive role in this respect. Conversely, collagen cross-linking could lead to an increase in fluorescence, and a correlation between age at death and fluorescence intensity should, therefore, also be taken into consideration.

In our collective, 6 of the 113 bones in the (shortest) PMI group 1 no longer showed UV- fluorescence and thus yielded a false-negative result. In the study by Hoke et al. [27], over 50% of all bones in the PMI range up to 60 years of age were also incorrectly determined as not forensically relevant. Consequently, a negative result (in terms of intensity) cannot, with certainty, rule out a forensically relevant PMI. In view of the legal consequences that would result from such a misjudgment, this level of uncertainty seems unacceptable.

In our investigations, the luminol test did not show a negative correlation between the degree of chemiluminescence of the luminol reaction and the macroscopically estimated PMI in our PMI group 1. Instead, it even showed a weakly positive correlation, which is quite surprising in view of previous study results. In previous studies, the luminol test has been described as a method that appears to be fundamentally capable of estimating the PMI, particularly in combination with other methods. Despite the long-established knowledge of the role of hemoglobin and its degradation products as catalysts of the luminol reaction [30], first described by Specht in 1937, it still does not seem entirely clear which components of bone samples examined with the luminol test cause chemiluminescence. In a study by Ramsthaler et al. [15], other examination methods such as the Hexagon-OBTI^Ⓡ^ test and the Combur^Ⓡ^ test were applied to bone samples, each of which yielded negative results for the presence of hemoglobin. Possible explanations for this finding are discussed in the aforementioned work. Irrespective of the question of which components in bone samples lead to the chemiluminescence of the luminol reaction, Ramsthaler et al.’s study yielded a weakly positive to strongly positive result in 14 of 16 samples in the critical PMI range of 10–100 years, a finding that is consistent with the results of our study. In addition, 30% of the bones with a PMI > 100 years (bones with a PMI > 1000 years included) in Ramsthaler et al.’s study yielded a positive luminol reaction. Based on other studies, the explanation for false-positive results may lie in numerous environmental factors that can cause positive chemiluminescence even though hemoglobin is no longer present in the bone [13, 31, 32]. In summary, these studies show that while the risk of a false-positive result is relevant, a negative result can, conversely, be seen as a good predictor for a PMI that is no longer forensically relevant [14–16, 19]. In this context, it should be noted that only a comparatively small number of bone samples with forensically relevant PMI were analyzed in the studies mentioned above. This stands in contrast to the number of bones with a forensically relevant PMI that were examined in our study. The explanation for the statistically significant correlation that was found in previous studies between the degree of chemiluminescence of the luminol method and the PMI may, therefore, be that sample collectives with sometimes very large PMI ranges, and thus also very old bone finds, were analyzed in those studies. In contrast, the present study was the first to analyze a larger number of bone samples with a more homogenous and shorter PMI range of up to 100 years with the luminol test. This circumstance may explain why no correlation between the degree of luminol chemiluminescence and PMI was found for our study collective.

However, it must be stressed that the exact PMI of the bone samples in our collective was not known and had only been estimated using macromorphological criteria. The fact that PMI estimates formed the basis for the classification of the luminol chemiluminescence intensity and the UV-fluorescence degree can be seen as a limitation of our study. Nonetheless, because of the quite well known lower and upper limit of the PMI, it can be seen as suitable for the present study, because it wasn’t the aim of the study to determine the exact PMI of the bones, but to compare the different methods.

## Conclusion

Based on the present results, it can be concluded that the extent of UV-fluorescence in the more recent PMI range of up to around 100 years is merely indicative of a forensically relevant PMI. In itself, UV-fluorescence does not provide a sufficiently valid assessment when used as a sole method. Similarly, the luminol test, alone, is also not an examination method that can be used to differentiate between forensically relevant and non-forensically relevant finds (meaning a PMI of over 50, but under 100 years). Nevertheless, it can still be assumed that a negative luminol test supports the assumption of a historical find. At least in combination with other methods, the luminol test is, therefore, still a suitable method for exclusion of a forensically relevant PMI.

## Data Availability

The data that support the findings of this study are available on request from the corresponding author.
